# Antioxidant Capacity of Carotenoid Extracts from the Haloarchaeon *Halorhabdus utahensis*

**DOI:** 10.3390/antiox12101840

**Published:** 2023-10-10

**Authors:** Ismene Serino, Giuseppe Squillaci, Sara Errichiello, Virginia Carbone, Lidia Baraldi, Francesco La Cara, Alessandra Morana

**Affiliations:** 1 Department of Experimental Medicine, University of Campania “Luigi Vanvitelli”, Via Costantinopoli 16, 80138 Naples, Italy; ismene.serino@unicampania.it; 2 Research Institute on Terrestrial Ecosystems, National Research Council of Italy (CNR), Via Pietro Castellino 111, 80131 Naples, Italy; giuseppe.squillaci@iret.cnr.it (G.S.); errichiellosara.se@icloud.com (S.E.); francesco.lacara@cnr.it (F.L.C.); 3 Institute of Food Sciences, National Research Council of Italy (CNR), Via Roma 64, 83100 Avellino, Italy; virginia.carbone@cnr.it; 4 Institute of Experimental Endocrinology and Oncology “Gaetano Salvatore”, National Research Council of Italy (CNR), Via S. Pansini 5, 80131 Naples, Italy; lidia.baraldi@cnr.it

**Keywords:** antioxidant, anti-hyaluronidase activity, bacterioruberin, carotenoids, haloarchaea, *Halorhabdus utahensis*, glucose, xylose, fructose, superoxide scavenging activity

## Abstract

Herein, we report on the production, characterization, and antioxidant power assessment of carotenoids from the haloarchaeon *Halorhabdus utahensis*. It was grown at 37 °C and 180 rpm agitation in halobacteria medium supplemented with glucose, fructose, and xylose, each at concentrations of 0.2%, 1%, and 2%, and the carotenoid yield and composition were investigated. The microorganism produced the carotenoids under all the conditions tested, and their amount followed the order glucose < xylose < fructose. The highest yield was achieved in 2% fructose growth medium with 550.60 ± 7.91 μg/g dry cell and 2428.15 ± 49.33 μg/L. Separation and identification of the carotenoids were performed by RP-HPLC and HPLC/APCI-ITMS^n^. Bacterioruberin was the main carotenoid detected and accounted for 60.6%, 56.4%, and 58.9% in 2% glucose, 1% xylose, and 2% fructose extracts, respectively. Several geometric isomers of bacterioruberin were distinguished, and representatives of monoanhydrobacterioruberin, and bisanhydrobacterioruberin were also detected. The assignment to *cis*-isomers was attempted through analysis of the UV/Vis spectra, intensity of *cis* peaks, and spectral fine structures. The extracts exhibited superoxide scavenging activity higher than butylhydroxytoluene, ascorbic acid, and Trolox, selected as antioxidant references. The anti-hyaluronidase capacity was investigated, and the 2% fructose extract showed the highest activity reaching 90% enzyme inhibition with 1.5 μg. The overall data confirm that *Hrd. utahensis* can be regarded as an interesting source of antioxidants that can find applications in the food and cosmetic sectors.

## 1. Introduction

Carotenoids are pigments from natural sources whose interest is due to their potential benefits for human health and possible industrial applications. In the last years, the interest from consumers towards the use of natural compounds in substitution of synthetic ones has increased and consequently, the demand for natural pigments as safe colorants in the food industry has increased [1]. Carotenoids are also significant for applications in the cosmetic industry because they can protect skin from sunlight damage, wrinkle development, and dryness [2]. These properties are due to their antioxidant action, but the antioxidant power also makes them capable of preventing human diseases associated with oxidative stress. Furthermore, carotenoids also show anti-inflammatory and anti-cancer effects [3,4]. 

The antioxidant power of carotenoids is strictly related to the chemical structure, in particular, the high number of conjugated double bonds that confers the capacity to act as radical scavengers toward free radical species responsible for damage to biological systems.

Carotenoids can be extracted from plants, but the variability in the growing conditions, as changes in climate or soil composition, have an influence on the yield. Chemical synthesis appears more effective but, even if this process provides pure compounds, it is also time consuming. In this context, microbial production of carotenoids seems more appealing and competitive because the growth conditions can be optimized in order to increase the carotenoid yield, and cheap substrates can be used as nutrients [5]. Once selected, the defined growth parameters can assure a standardized production of the pigmented molecules.

Several bacteria and yeasts are carotenoid producers, such as the dibenzothiophene desulfurizing bacterium *Gordonia alkanivorans* strain 1B, which synthesizes lutein, canthaxanthin, and astaxanthin, and the yeast *Rhodotorula mucilaginosa* with beta-carotene, torularhodin, and torulene among its main carotenoid components [6,7]. A detailed list of carotenoid-producing microorganisms and their biosynthesized pigments is available in the carotenoid database at the link: http://carotenoiddb.jp/index.html (accessed on 10 July 2023).

Many microbial carotenoids are of intracellular origin and an effective method of cell disintegration must be foreseen for their recovery. Usually, bacterial cells are easier to disrupt in comparison to microalgae and yeasts that possess a cell wall that is hard to disintegrate [8]. However, intracellular carotenoids can be strictly associated with other cell components, such as proteins, making the extraction process more complex and time consuming [9]. Carotenoids can also be membrane bound, as in the case of several red-pigmented halophilic microorganisms. The production of these pigments is responsible for the pink coloring of the habitats where they live, such as Retba Lake in Senegal and Lake Natron in Tanzania. These carotenoids act as membrane stabilizers by increasing the membrane rigidity. Moreover, they provide protection against UV rays when microorganisms are exposed to sunlight [10]. Halophilic microorganisms are present in all three domains of life: Archaea, Bacteria, and Eukarya, and are able to live in environments characterized by high salt concentrations. Extreme halophiles show optimal growth in media with NaCl from 15% to 30%, and the main representatives of this group are the halophilic aerobic Archaea (also called haloarchaea) [11]. The high salt amount required for their growth makes halophiles advantageous to cultivate because they allow non-sterile conditions.

Bacterioruberin (BR), a C50 xanthophyll, is the characteristic and main representative of pigments produced by haloarchaea. Its precursors monoanhydrobacterioruberin (MABR) and bisanhydrobacterioruberin (BABR) are also synthesized by the majority of halophilic Archaea, but other compounds, such as lycopene, phytoene, and beta-carotene, can be produced [12].

*Halorhabdus utahensis* is an extreme haloarchaeon belonging to the family *Halobacteriaceae*. It was isolated from sediment from the Great Salt Lake (Utah, USA) by Wainø et al., in 2000 [13]. The microorganism shows a pleomorphic shape and requires an optimal NaCl concentration of 27% for its growth. The red-pigmented colonies observed after 3 weeks of incubation indicated the capability of *Hrd. utahensis* of producing carotenoids.

With the aim of finding novel carotenoid-producing microorganisms to use as antioxidant sources, the present paper reports on the production of carotenoids from *Hrd. utahensis* grown in culture medium with different sugars added. The carotenoids were identified, and the extracts were tested for their antioxidant activity. Furthermore, the inhibition of the hyaluronidase activity was measured in order to verify the suitability of the carotenoids as active ingredients in preparations intended for skin care. To the best of our knowledge, this is the first report about the identification and assessment of the antioxidant power of carotenoids from the genus *Halorhabdus.*

## 2. Materials and Methods

### 2.1. Chemicals

Chemicals for the preparation of the growth medium, 2,2-diphenyl-1-picrylhydrazyl(DPPH), pyrogallol, Tris-HCl, EDTA-Na_2_, 2,4,6-tripyridyl-S-triazine (TPTZ), FeCl_3_·6H_2_O, ascorbic acid, butylhydroxytoluene (BHT), Trolox, hyaluronic acid (HA) sodium salt from *Streptococcus equi*, hyaluronidase (HAase) from bovine tests, and 4-(dimethylamino)benzaldehyde (DMAB) were purchased from Sigma-Aldrich Co. (Milano, Italy). High-performance liquid chromatography (HPLC)-grade methanol and ethyl acetate were obtained from Fisher Chemical (Fisher Scientific Italia, Rodano, Milan, Italy). HPLC-grade water (18.2 mΩ) was prepared using a Millipore Milli-Q purification system (Millipore Corp., Bedford, MA, USA).

### 2.2. Hrd. utahensis Cultivation

*Hrd. utahensis* (DSM-12940), obtained from Deutsche Sammlung von Mikroorganismen und Zellkulturen (DSMZ, Braunschweig, Germany), was revitalized following the manufacturer’s instructions. Then, the microorganism was grown aerobically in medium 927 (M927, DSMZ catalogue) at 37 °C and 180 rpm in a rotary water bath shaker (Aquatron, Infors AG, Bottmingen, Switzerland) and stored in the same medium containing 15% (*v*/*v*) glycerol at −80 °C until used (starter culture). The starter culture was added to 20 mL of the growth medium in a 100 mL Erlenmeyer flask. At the exponential phase, an aliquot was used to inoculate a 500 mL Erlenmeyer flask containing 100 mL of growth medium in order to obtain an initial optical density (OD) of 0.150. The growth was gradually scaled up to 500 mL in a 2 L Erlenmeyer flask. Glucose, fructose, and xylose were added to the culture medium as the carbon source at 2, 10, and 20 g/L. The archaeal growth was followed by measuring the OD at 600 nm (Thermo Scientific spectrophotometer, Genesys 180 model, Rodano, Milan, Italy). When the stationary phase was reached, cells were harvested by centrifugation at 9220 rpm for 40 min at 4 °C (Sorvall, RC6+). The cell-free supernatant was discarded, and the biomass was stored at −20 °C until used.

### 2.3. Extraction and Quantification of Carotenoids

The frozen cells were thawed and treated with methanol for the extraction of carotenoids, according to Squillaci et al., with some modifications [14]. Briefly, 500 mg of wet cells was mixed with 1 mL of methanol containing 0.5% BHT and stirred for 60 min in order to extract the membrane-bound carotenoids. The suspension was centrifuged at 13,200 rpm for 20 min at 4 °C, and the colored supernatant (first extract) was recovered. A second extraction was performed by mixing the cells with 2 mL of methanol/0.5% BHT for 60 min and centrifuging, as reported above. The colored supernatant (second extract) was separated from the slightly colored cells. The third extract was obtained by adding 1 mL of methanol/0.5% BHT to the cells. After stirring for 60 min, the mixture was centrifuged. The colored supernatant was added to the previous extracts and the colorless cells were discarded. All the extraction steps were performed on ice and maintained in the dark to avoid isomerization phenomena. The amount of carotenoids contained in the whole extracts was calculated according to Scott [15]. The extinction coefficient of BR in methanol (A_1cm_^1%^ = 2660) was used, and the quantity of carotenoids was expressed as micrograms per gram of dry cells [16].

### 2.4. Analysis of the Extracts

UV/Vis spectra of the whole extracts were recorded between 300 and 600 nm by means of a spectrophotometer Genesys 180.

The analysis of the carotenoids was carried out by reverse-phase high-performance liquid chromatography (RP-HPLC) using a Dionex Ultimate 3000^®^ HPLC system equipped with a quaternary pump and a diode array detector (Dionex, Sunnyvale, CA, USA) as follows: aliquots of extracts (50 μL) were injected into a Luna C18 (2) column (250 × 4.6 mm, 5.0 μm) equipped with a SecurityGuard™ pre-column containing a C18 cartridge (Phenomenex Inc. Castel Maggiore, Bologna, Italy). The separation was performed at a flow rate of 1 mL/min by applying the following linear gradient: from 0 to 100% solvent B in 45 min, 100% solvent B for a further 10 min, and from 100% solvent B to 100% solvent A in 5 min. The mobile phase was degassed ultrapure water/methanol (1:9 *v*/*v*) (solvent A) and ethyl acetate/methanol (1:9 *v*/*v*) (solvent B). Peak elution was monitored at 490 nm, and the online spectrum of each peak was registered between 300 and 600 nm. The percentage of each carotenoid detected in the extracts was determined by dividing the area of the carotenoid by the sum of the areas of all identified carotenoids.

### 2.5. HPLC/UV-Vis and HPLC-Atmospheric Pressure Chemical Ionization Multistage Ion Trap Mass Spectrometry (APCI-ITMS^n^) Analyses

The carotenoid extract from *Hrd. utahensis* grown in M927 with added 2% glucose was also analyzed by HPLC/UV-Vis and HPLC/APCI-ITMS^n^ on a Surveyor MS micro HPLC with a diode array detector and coupled with an LCQ DECA XP Max ion trap mass spectrometer, equipped with Xcalibur^®^ system manager data acquisition software (Thermo Finnigan, San Jose, CA, USA). The carotenoids were separated on the Luna C18 (2) column previously described, at a flow rate of 0.5 mL/min, by using the previous chromatographic conditions with slight modifications: from 0 to 100% solvent B in 90 min, 100% solvent B for further 10 min, from 100% solvent B to 100% solvent A in 5 min. The column effluent was split into two by a “T junction” placed after the chromatographic column and analyzed “online” by UV/Vis and APCI/MS; a total of 80% of the effluent was sent to the UV/Vis detector (detection at 490 nm), while 20% of the effluent was analyzed by APCI/MS in positive ion mode. The APCI vaporizer temperature was set at 450 °C, the capillary voltage at 13 V, the discharge current at 5 μA, and the tube lens offset at −15 V. The capillary temperature was 250 °C. Data were acquired in MS, MS/MS, and MS^n^ scanning modes and recorded in the 400–2000 *m*/*z* range. The total ion current (TIC) profile was produced by monitoring the intensity of all the ions produced during the chromatographic run and acquired for every scan.

### 2.6. Antioxidant Assays

The antioxidant capacity was assessed for carotenoid extracts from *Hrd. utahensis* grown in M927 with added 2% glucose, 2% fructose, and 1% xylose. The following assays were carried out using extracts prepared with methanol in the absence of BHT.

#### 2.6.1. Radical Scavenging Activity

RSA was estimated by measuring the discoloration of the stable purple free radical 2,2-diphenyl-1-picrylhydrazyl (DPPH^●^) in accordance with Jiménez-Escrig et al. [17]. An opportune volume of extracts containing 5 μg of carotenoids was diluted to 150 μL with methanol and mixed with 100 μM DPPH^●^ (1.35 mL). A negative control was prepared with 150 μL of absolute methanol in 100 μM DPPH^●^ (1.35 mL). The reaction mixture was prepared in a cuvette; then, the cuvette was placed in the spectrophotometer, and the discoloration reaction of the free radical was followed at 580 nm for 30 min against a blank containing absolute methanol (1.5 mL). The RSA % was calculated according to the following formula:

RSA % = (Absorbance_negative control_ − Absorbance_sample_/Absorbance_negative control_) × 100

where Absorbance_negative control_ and Absorbance_sample_ were the values recorded at 30 min (reaction plateau). These values were compared with those obtained by analyzing 5.0 μg of ascorbic acid, BHT, and Trolox as positive controls.

#### 2.6.2. Superoxide Scavenging Activity

The SSA was assessed by the pyrogallol autoxidation method described by Li with minor modifications [18]. An appropriate volume of extracts containing 5 μg of carotenoids was mixed in a quartz cuvette with 50 mM Tris-HCl (pH 7.4) containing 1 mM EDTA-Na_2_ buffer solution (final volume 983 μL). Then, 60 mM pyrogallol in 1 mM HCl (17 μL) was added to the above solution. After quick stirring, the absorbance was recorded at 325 nm every 30 s for a total of 300 s against a blank containing the buffer solution (1 mL). As a control sample, a buffer solution (983 μL) containing 60 mM pyrogallol in 1 mM HCl (17 μL) was prepared and subjected to absorbance measurement at 325 nm as reported above. The scavenging ability was calculated according to the following formula:

SSA % = (Absorbance_control_ − Absorbance_sample_/Absorbance_control_) × 100

where Absorbance_control_ and Absorbance_sample_ were the values recorded at 300 s (reaction plateau). These values were compared with those obtained with 5.0 μg of ascorbic acid, BHT, and Trolox as positive controls.

#### 2.6.3. Ferric Reducing Antioxidant Power

The FRAP assay was performed according to Benzie and Strain [19]. Briefly, the FRAP solution was prepared and mixed in a 10:1:1 (A:B:C) ratio at the time of use; the FRAP solution contained the following reagents: 0.3 M sodium acetate buffer, pH 3.6 (A), 0.01 M 2,4,6-tripyridyl-S-triazine (TPTZ) in 0.04 M HCl (B), and 0.02 M FeCl_3_·6H_2_O (C). A proper volume of extracts containing 5 μg of carotenoids was diluted to 50 μL with methanol and added to the FRAP solution (1.5 mL). After a 4 min incubation, the absorbance was read at 593 nm against a blank made of FRAP solution. The ferric-reducing power was compared with the three antioxidants chosen as positive controls: ascorbic acid, BHT, and Trolox.

The FRAP values of the samples were converted into the corresponding micrograms of ascorbic acid, BHT, and Trolox and expressed as antioxidant equivalents according to the following dose–response curves:

1. A593nm = 0.1572 μg r^2^ = 0. 9998 for ascorbic acid;
2. A593nm = 0.0026 μg r^2^ = 0.9945 for BHT;
3. A593nm = 0.0994 μg r^2^= 0.9914 for Trolox.

The quantities used ranged from 0.25 to 5.0 μg for ascorbic acid, from 5.0 to 200.0 μg for BHT, and from 1.0 to 7.0 μg for Trolox.

### 2.7. HAase Inhibition Assay

The HAase inhibition was assessed according to Ferreres et al., with some modifications [20]. The reaction mixture was prepared by addition of the reagents in the described order: 100 μL of 200 mM sodium formate buffer (pH 3.68) containing 100 mM NaCl and 0.2 mg/mL BSA, 0.25, 0.50, 1.00, and 1.50 μg of carotenoid extracts diluted to 100 μL with methanol, 200 μL of HA solution in water (5 mg/mL), and 50 μL of HAase in 0.9% NaCl (3600 U/mL). The reaction mixture was incubated at 37 °C for 1 h, stopped by adding 1 M Na_2_CO_3_ (25 μL), and followed by immersion in boiling water for 3 min. After cooling on ice, 750 μL of DMAB solution (2 g of DMAB in 2.5 mL of 10 M HCl and 17.5 mL of glacial acetic acid diluted 1:2 with glacial acetic acid before use) was added, followed by incubation at 37 °C for 20 min. The absorbance of the purple solution was recorded at 560 nm, and the HAase inhibition percentage was calculated according to the following formula:

HAase inhibition % = (Absorbance_HAase_ − Absorbance_sample_/Absorbance_HAase_) × 100

HAase activity in absence of the carotenoid extracts was taken as 100% for calculating the inhibition.

### 2.8. Statistical Analysis

Analyses were performed in triplicate and expressed as mean standard deviation (SD) calculated by Microsoft Excel. Experimental data were analyzed using GraphPad Prism (version 5). Significant differences were determined by one-way analysis of variance (ANOVA) completed by Bonferroni post-tests. Mean values were considered significantly different at *p* ≤ 0.05.

## 3. Results and Discussion

### 3.1. Influence of Sugars on Carotenoid Production, Extraction, and Quantification

**Figure 1:**
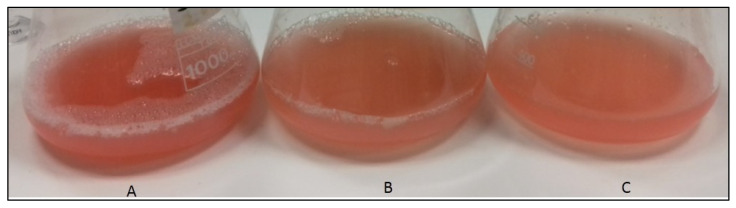
*Hrd. utahensis* growths in M927 added with 2% fructose (**A**), 1% xylose (**B**), and 2% glucose (**C**).

Cultivation of *Hrd. utahensis* was performed in M927 with added glucose, fructose, and xylose as carbon sources. No studies about optimized conditions were carried out at this stage, as our purpose was only to compare the carotenoid yield in relation to the different sugars used. Three sugar concentrations were selected (2, 10, and 20 g/L) for the following reasons: 2 g/L is the concentration indicated in the recipe for medium 927 (DSMZ catalogue), 10 g/L is a common sugar concentration used in growth media [21,22], and 20 g/L was chosen to evaluate whether a higher concentration favored a greater production of biomass and consequently, of carotenoids. All sugars allowed the microorganism growth except 2% xylose (Figure 1).

Even if the stationary phase was reached at different times depending on the type of sugar and concentration in the culture medium, the microorganism was able to produce carotenoids with all the sugars tested. After the complete extraction of carotenoids with methanol, pink–red extracts and colorless biomasses were obtained.

**Figure 2:**
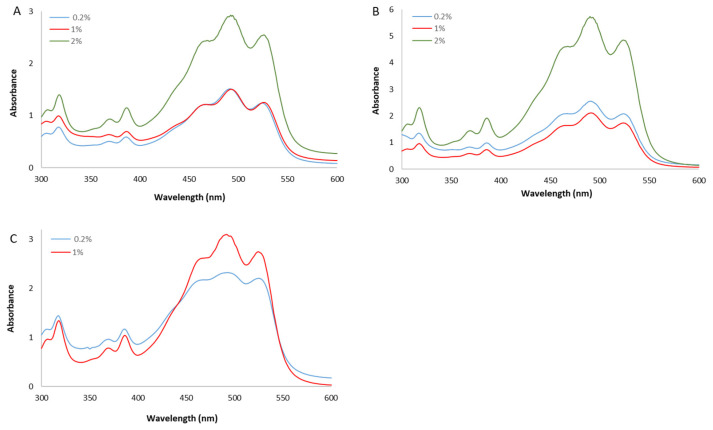
UV/Vis spectra of whole methanol extracts from *Hrd. utahensis* grown in M927 added with glucose (**A**), fructose (**B**), and xylose (**C**) at different concentrations.

The spectra of the whole extracts, recorded between 300 and 600 nm, presented the characteristic shape of the red carotenoid molecules with the three-fingered peaks showing maximum absorptions at 468, 490, and 524 nm (Figure 2).

The amount of carotenoids was different in relation to the sugar added in the culture medium (Table 1). Statistically significant differences were measured among the carotenoid yields following the order glucose < xylose < fructose (*p* < 0.0001). The tested concentrations of xylose had no significant effects on the carotenoid yield per g of dry cells because similar quantities of pigments were measured (*p* > 0.05). No statistically significant differences were also observed between carotenoid yields of 0.2% and 1% glucose (*p* > 0.05).

As the biomass yield was affected by the concentration of the carbon source, the content of pigments per liter of culture medium increased from 0.2% to 2% with all sugars investigated (1% for xylose). The lowest yield was obtained when *Hrd. utahensis* was grown in 0.2% glucose (110.36 ± 5.05 μg/g dry cell and 226.24 ± 14.64 μg/L), whereas 2% fructose provided the highest amount with 550.60 ± 7.91 μg/g dry cell and 2428.15 ± 49.33 μg/L. Few species of the genus *Halorhabdus* have been described up to now; among them, the *Hrd. rudnickae* strains WSM-64^T^ and WSM-66 form red-pigmented colonies beyond *Hrd. utahensis*, but the latter represents the first species of this genus whose carotenoids have been identified [23].

According to the present results, *Hrd. utahensis* can be considered a good carotenoid producer because the amount of pigments synthesized was comparable to or higher than that produced by other haloarchaea described so far. In fact, the most cited haloarchaeon commonly used as a model microorganism, *Haloferax mediterranei*, produced 760 μg/L of carotenoids, whereas *Halorubrum* sp. HRM-150, and the recently isolated *Haloterrigena thermotolerans* strain K15 provided a carotenoid yield of 81.50 and 21.43 μg/g dry biomass, respectively [24,25,26].

The influence of different sugars on carotenoid production in *Haloarchaea* has been poorly investigated so far. Some studies described comparisons among sugars and compounds of different nature, such as the work of Vázquez-Madrigal et al. [22]. Glucose, succinic acid, and glutamic acid affected in a different manner the growth and carotenoid production of three *Haloarchaea* isolated from Sonora Saltern, Mexico. Glucose enhanced the biomass and carotenoid yields of *Haloarcula* sp. M1 and *Halorubrum* sp. M5 with respect to the control medium, whereas glutamic and succinic acids did not affect the carotenoid production of the three isolated. Giani et al. [27] explored the influence of diverse carbon sources on the biomass and carotenoid yields of *Hfx. mediterranei* and studied the antioxidant, antiglycemic, and antilipidemic properties of the pigment extracts. Glucose and starch were selected as sugars to be tested, and both were effective in promoting cell growth and carotenoid production at similar levels.

An explanation for the different carotenoid yields obtained with the sugars tested in this present paper is hard. *Haloarchaea* synthesizes carotenoid precursors through the mevalonate pathway that leads to lycopene, which in turn forms BR. This biosynthetic pathway starts with acetyl-CoA that is obtained from glycolysis [28]. It can only be assumed that, since the different sugars are inserted at different points during glycolysis, they could have a diverse influence on the production of carotenoids.

### 3.2. Analysis Identification, and Quantification of Carotenoids

The separation and identification of carotenoids were carried out by RP-HPLC/UV–Vis analyses of the extracts obtained from 2% glucose, 2% fructose, and 1% xylose biomasses, as they provided the highest quantity of pigments for each sugar tested.

**Figure 3:**
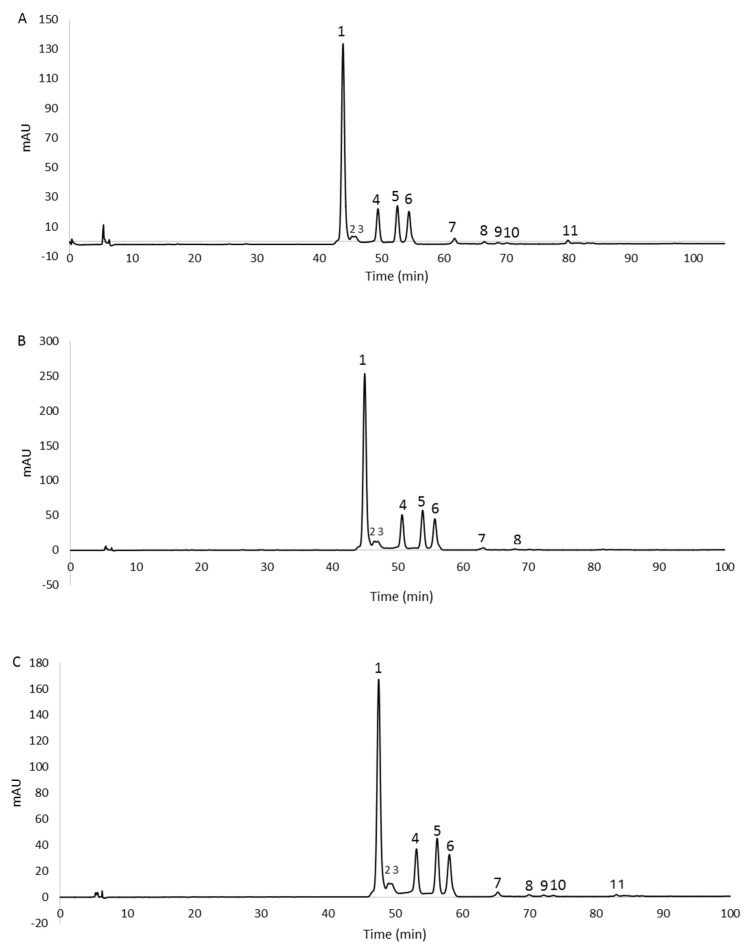
RP−HPLC chromatograms, recorded at 490 nm, of methanol extracts from 2% glucose (**A**), 2% fructose (**B**), and 1% xylose (**C**) biomasses. For peaks identification, see Table 2.

**Figure 4:**
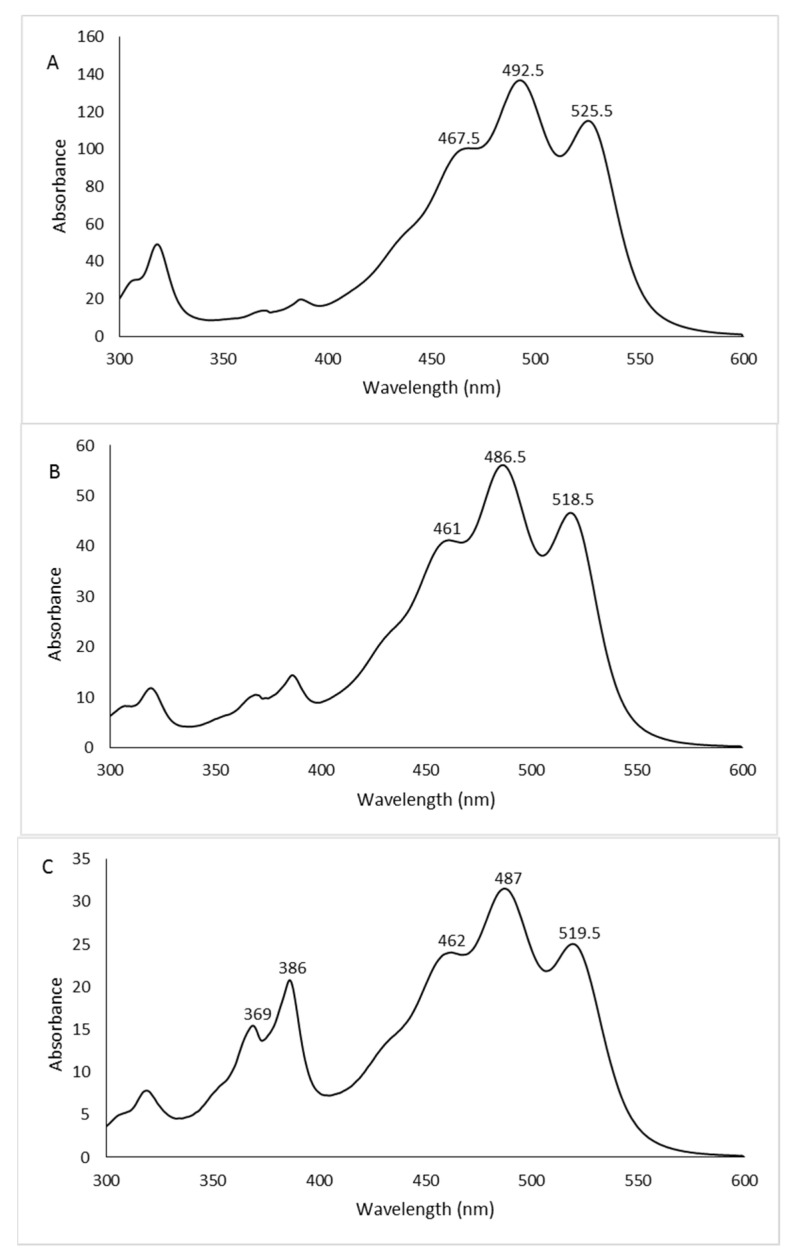
UV/Vis absorption spectra of some representative peaks: 2% glucose peak 1 (**A**), 2% fructose peak 5 (**B**), 1% xylose peak 6 (**C**). See Figure 3 for peak numbers.

The chromatographic profiles are shown in Figure 3. The peaks, detected at 490 nm, were identified as carotenoids by means of spectral analysis, which revealed the typical three-fingered shape. Figure 4 shows some UV/Vis absorption spectra of the single peaks resolved by RP-HPLC.

Eleven, eight, and eleven peaks were detected in the 2% glucose, 2% fructose, and 1% xylose extracts, respectively. In order to confirm the identity of the carotenoids, the 2% glucose extract was also analyzed by HPLC/APCI-ITMS^n^. This extract was selected because it contained all the peaks detected in the three extracts. Carotenoids were tentatively identified from their pseudomolecular [M + H]^+^ ions, together with the interpretation of their collision-induced dissociation (CID) fragments and from their UV/Vis spectra. In particular, mass spectrometric analysis revealed that peaks 1–6 showed the same pseudomolecular ion [M + H]^+^ at *m*/*z* 741.5, consistent with the BR isomers. Similarly, peaks from 7 to 10 exhibited a [M + H]^+^ ion at *m*/*z* 723.5, and peak 11 showed a [M + H]^+^ ion at *m*/*z* 705.5, corresponding to different isomers of the BR derivatives MABR and BABR, respectively. Furthermore, all these compounds were submitted for tandem and multistage mass spectrometric analyses, showing a similar fragmentation pattern under positive ion APCI conditions, with losses in accordance with those previously reported [14]. As the mass spectrometric analyses did not lead to differentiation among the isomers, the assignment of the structures was carried out, where possible, through the analysis of their UV/Vis spectra. In detail, the maximum wavelengths corresponding to the trident peaks were considered for each spectrum and, where present, those relating to the *cis* peaks were analyzed in order to evaluate their intensity (%A_B_/A_II_) and the spectral fine structure (% III/II) for corroborating the assignment of the isomers [29]. The list of the identified carotenoids is reported in Table 2.

The results obtained from the mass spectrometric analysis of the 2% glucose extract, and the comparison of UV/Vis spectra between the same peaks of the three samples allowed the identification of the carotenoids also present in the fructose and xylose extracts. They are indicated in Table 3.

All the identified carotenoids belong to BR or the precursors MABR and BABR, and representatives of the BR family (BR and related isomers) were the most abundant. The main peak detected (peak 1, Figure 3A–C) corresponded to all-*trans*-BR accounting for 60.6%, 58.9%, and 56.4% of the total carotenoids measured in 2% glucose, 2% fructose, and 1% xylose samples, respectively. Five *cis* isomers of BR were identified in the three extracts thanks to the shift of the λmax related to the all-*trans*-BR trident peaks to shorter wavelengths (hypsochromic effect). These isomers accounted for 35.7%, 39.9%, and 41.3% in 2% glucose, 2% fructose, and 1% xylose extracts, respectively.

The peaks numbered 3 (Figure 3A–C) were not assigned to a specific isomer even if the absorption intensity of their *cis* peaks in the spectra (% A_B_/A_II_ value) suggested the presence of isomers whose double bonds were positioned in the internal part of the carbon chain. This assumption was based on Zechmeister’s statement that the absorption intensity of the *cis* peak increases when the double bond is closer to the center of the molecule [30].

The all-*trans*-MABR was identified in all extracts together with the isomer 5-*cis*-MABR, whereas the 9-*cis*-MABR, 13-*cis*-MABR, and all-*trans*-BABR were detected in 2% glucose and 1% xylose extracts. Only one double *cis* isomer was detected, and precisely the 5-*cis*,9’-*cis*-BR. It was present in a very small amount, ranging from 1.92% (2% glucose extract) to 2.73% (1% xylose extract). Double *cis* isomers are not widespread; in fact, their presence is described in only a few extreme halophilic microorganisms. The carotenoid 5-*cis*,9’-*cis*-BR was previously identified in the *Hfx. volcanii* and *Htg. turkmenica* [14,31], whereas 9-*cis*,9’-*cis*-BR was detected in the *Htg. thermotolerans* strain K15 and *Hfx. volcanii* [26,31]. Lizama et al. [32] identified the double *cis* isomers 5-*cis*,26-*cis*-BR and 9-*cis*,26-*cis*-BR in several strains related to the genus *Haloarcula* isolated from the saline Tebenquiche lake in the Atacama Desert (Chile), but although these isomers were described for the first time, no spectral data were reported to confirm the assignment.

The presence of BR, MABR, and BABR in the extracts from *Hrd. utahensis* was in agreement with data previously reported in the literature; in fact, MABR and BABR were already identified in other halophilic microorganisms, such as the *Har. japonica* strain TR-1 and *Hfx. Mediterranei* strain R4 [10,33]. However, the qualitative composition of the carotenoid extracts can be different among halophilic Archaea as ascertained by the analysis carried out on the following microorganisms: *Halogeometricum* sp. strain ME3, *Haloferax* sp. strain ME16, and *Haloarcula* sp. strain BT9. In fact, among the pigments identified, BABR was detected in *Halogeometricum*, MABR in *Haloferax*, and trisanhydro-BR in *Haloarcula* [34].

It is noteworthy that, despite these differences, BR was always present in such microorganisms as well as in all haloarchaea described so far, thus demonstrating that this pigment is the main carotenoid among those identified in the extreme halophiles. It has important biological roles, such as a protecting agent against UV and gamma-ray radiations and as a cell membrane stabilizer because its quantity increased when *Hfx. volcanii* was subjected to osmotic stress in a culture medium with low salt concentration [35,36].

However, BR and precursors do not represent C50 carotenoids that are peculiar to halophiles, as they were also identified in some non-halophilic species, such as the psychotrophic bacterium *Micrococcus roseus,* isolated from soil samples in Antarctica, where BR and three *cis*-isomers were detected [37]. In addition, Flegler and Lipski recently described the presence of BR in the pink-pigmented species *Arthrobacter agilis* DSM 20550T and *A. bussei* DSM 109896T [38].

### 3.3. Estimation of the Antioxidant Power

Due to the great industrial potential of BR, whose antioxidant capacity is higher than other carotenoids, such as astaxanthin and beta-carotene [10,25], antioxidant tests were performed using *Hrd. utahensis* carotenoid extracts for assessing their antioxidant capacity. Three diverse assays were chosen because the antioxidant activity can depend on several modes of action, and the results were compared with the antioxidant power of the following reference compounds: BHT, ascorbic acid, and Trolox.

The DPPH radical scavenging assay was used to investigate the ability as radical scavengers of the carotenoid extracts. This method is widely considered as the first approach for evaluating antioxidant activity of a molecule or a blend of compounds thanks to its low cost, speed of preparation, and ease of execution. In fact, the DPPH assay has been adopted as the official method for the determination of the antioxidant power in beverages and foods by the AOAC (Association of Official Agricultural Chemists’) [39].

**Figure 5:**
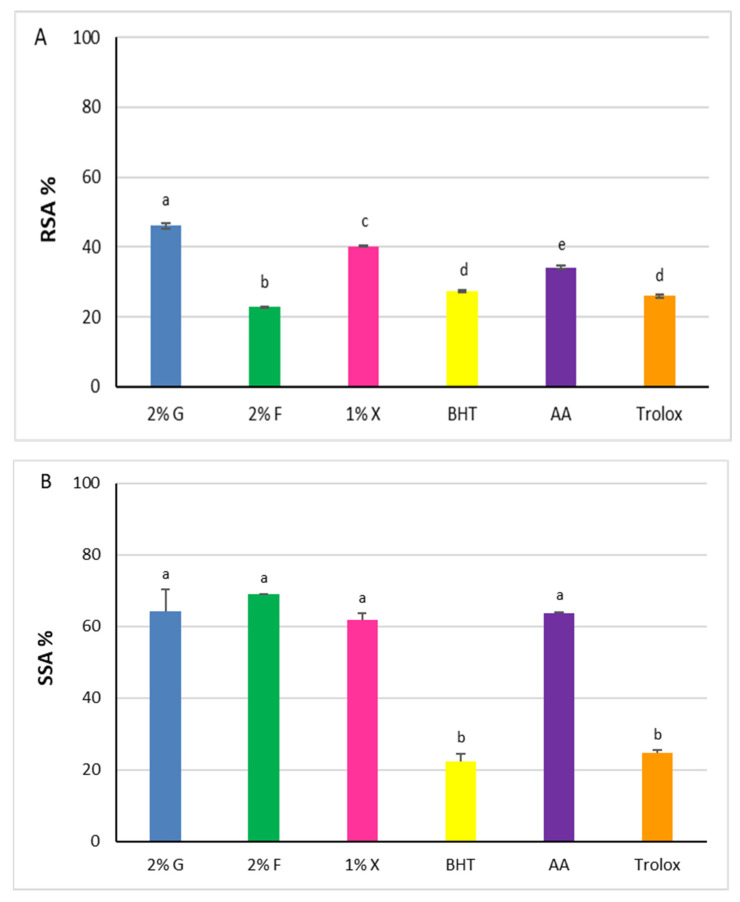
Antioxidant power of carotenoid extracts from *Hrd. utahensis*. Radical Scavenging Activity (RSA %) (**A**), Superoxide Scavenging Activity (SSA %) (**B**). 2% G = 2% glucose; 2% F = 2% fructose; 1% X = 1% xylose; BHT = butylhydroxytoluene; AA = ascorbic acid. Bars with different letters denote significant statistical differences at *p* < 0.0001.

The three extracts showed antioxidant capacity when tested using the DPPH assay, with RSA% values increasing as the reaction time increases, reaching the plateau after 30 min. Results at 30 min with 5 μg of carotenoids are shown in Figure 5A.

Statistically significant differences were recorded among the extracts (*p* < 0.0001), with the RSA% increasing in accordance with the following order: 2% fructose < 1% xylose < 2% glucose. The values of glucose and xylose extracts, 46.1% ± 0.8 and 40.1% ± 0.1, respectively, were higher than the antioxidants chosen as comparison molecules (*p* < 0.0001), whereas the radical scavenging power of 2% fructose extract was lower (22.8% ± 0.1).

The superoxide radical anion (O_2_^•−^) is one of the ROS generated in the human body responsible for oxidative harmful effects. Carotenoids can react with the superoxide radical anion, performing their function as antioxidants through an electron transfer reaction [40]. According to this, the SSAs of the carotenoid extracts from *Hrd. utahensis* were estimated by an assay based on the inhibition of the pyrogallol autoxidation. The assay produces the superoxide radical anion, which is directly involved in the autoxidation reaction [41] and whose amount can be easily measured at 325 nm. When an antioxidant is added to the reaction mixture, it reacts with the superoxide radical anion which is no longer available for the autoxidation reaction. As a consequence, the absorbance measured is lower than in the absence of antioxidants. All extracts were able to reduce the production of the superoxide radical anion, showing comparable antioxidant effects (Figure 5B). The differences measured among the SSA% values were not statistically significant (*p* > 0.05) ranging from 61.8% ± 1.9 (1% xylose) to 68.9% ± 0.2 (2% fructose). The extracts tested exhibited antioxidant powers higher than BHT and Trolox (22.2% ± 2.2 and 24.8% ± 0.7, respectively) (*p* < 0.0001) but slightly higher or comparable to ascorbic acid (63.6 ± 0.3) (*p* > 0.05).

The principle of the FRAP assay is based on the ability of antioxidants to reduce the Fe(III) 2,4,6-tripyridyl-s-triazine complex to the Fe(II) 2,4,6-tripyridyl-s-triazine complex in an acid environment. As the reduced complex is colored, the reaction can be monitored at the maximum absorption wavelength of 593 nm by a spectrophotometer. The absorbance of the three extracts measured after 4 min of assay was converted into micrograms of BHT, ascorbic acid, and Trolox by the corresponding calibration curves (Table S1) and expressed as antioxidant standard activity equivalents (Table 4). The extracts showed ferric reducing antioxidant power to different extents with statistically significant differences (*p* < 0.001) among them in relation to each antioxidant standard considered. Two percent glucose and one percent xylose were less antioxidant than ascorbic acid and showed statistically significant differences between them (*p* < 0.0001). All three extracts exhibited a higher antioxidant power than BHT since the micrograms of BHT necessary to reach the same FRAP response obtained with 5 micrograms of carotenoids were 36.6, 65.7, and 55.6 times greater than the 2% glucose, 2% fructose, and 1% xylose, respectively. The differences measured among the BHT equivalents of the three extracts were statistically significant (*p* < 0.001). Two percent fructose and one percent xylose also showed higher antioxidant activity than Trolox with differences in Trolox antioxidant equivalents statistically significant (*p* < 0.001). On the contrary, the 2% glucose extract was less antioxidant than Trolox, showing significant differences in the other two extracts (*p* < 0.0001).

The overall results indicated that the extracts exerted their antioxidant power towards all the assays selected, thus confirming the ability of *Hrd. utahensis* to provide antioxidant agents acting by transfer of electrons to radical species or to metals with high oxidation numbers. Definitely, the extract from the 2% fructose exerted the highest antioxidant power with the SSA and FRAP assays, whereas the 2% glucose extract had the strongest RSA.

A substantial uniformity in the carotenoid composition was detected in the extracts. In all of them, the major representative was the BR family (all-*trans*-BR and isomers) accounting for 96.3%, 98.8%, and 97.7% in the 2% glucose, 2% fructose, and 1% xylose extracts, respectively. It is conceivable that the differences observed in the antioxidant behavior could depend on other compounds present in the extracts and not detectable under our experimental conditions.

Comparison with the antioxidant power of extracts from other halophiles is not easy because the test conditions are often different. For example, for determining the RSA value, the concentration of the DPPH reagent can vary among diverse authors, the wavelength of the assay can be 517 nm or 580 nm, and the reagent volumes may also differ. For this reason, the results are usually compared with those of reference antioxidants [42,43]. In addition, thiss paper reported for the first time the SSA determination of carotenoids from extreme halophilic microorganisms; therefore, only a comparison with some positive controls is possible.

BHT, known as European additive E321, was selected as the positive control because it is currently used as an antioxidant ingredient in fat and oil-rich cosmetics and food. However, as it is a synthetic molecule whose safety is in question [44], the search for antioxidants from natural sources with comparable or higher power is very active. Ascorbic acid was selected as a positive control of natural origin. It is known as E300 and is used as an additive in food products thanks to its powerful antioxidant action [45]. Trolox, a derivative of Vitamin E, was selected because it is widely used as a positive control for measuring the antioxidant power of blends of molecules by means of several assays [46,47].

### 3.4. HAase Inhibition

**Figure 6:**
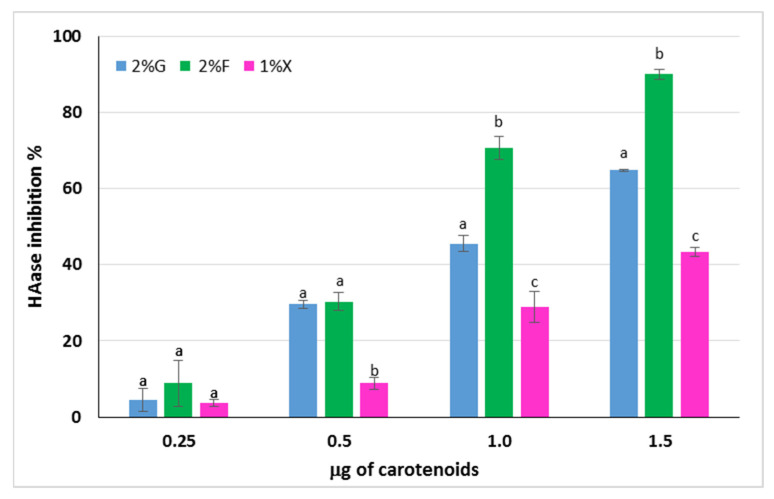
Inhibitory activity of carotenoid extracts from *Hrd. utahensis* towards HAase. 2% G = 2% glucose; 2% F = 2% fructose; 1% X = 1% xylose. Bars with different letters within each group with the same carotenoid amount denote significant statistical differences at *p* < 0.001.

Hyaluronic acid (HA) is a polysaccharide produced by fibroblasts and is responsible, together with collagen, for the elasticity of human skin. It is a component of the extracellular matrix that can undergo degradation by the action of ROS or by means of hyaluronidase enzymes (HAases), whose expression is increased in the case of oxidative stress [48,49]. The ability to inhibit the HAase action by the carotenoid extracts from *Hrd. utahensis* was investigated. The extracts were able to inhibit the enzyme action to different extents, and for all of them, a directly proportional dose–response relationship was observed (Figure 6). Differences in the anti-hyaluronidase activity of the extracts were statistically significant at 1.0 and 1.5 μg tested (*p* < 0.001), whereas the effects were comparable at 0.25 μg (*p* > 0.05). The 2% fructose extract showed the strongest HAase inhibitory effect, reaching 90.0% ± 0.9 with 1.5 μg. The HAase inhibition percentage varied from 4.5% ± 3.1 to 64.9% ± 0.3 for the 2% glucose extract, whereas the 1% xylose was the least active towards the enzyme with 43.3% ± 1.2 inhibition as the maximum value reached.

To the best of our knowledge, the present paper reports for the first time the inhibition activity of carotenoids from extreme halophilic Archaea against HAase. Some papers have already described the HAase inhibition action of carotenoids, such as Morone et al. [50] and Kurinjery et al. [51], but the pigments were extracted from seven cyanobacteria strains isolated in Portugal, and from several brown seaweeds, respectively.

## 4. Conclusions

In the present study, the production of carotenoid blends with high antioxidant power was achieved by the extreme halophilic Archaeon *Hrd. utahensis* grown in media with different added sugars. Glucose, fructose, and xylose were selected as the carbon sources because wastes containing the mentioned sugars can be easily obtained in huge amounts from industrial processing of fruits and lignocellulosic biomasses. In this optic, it would be possible to achieve two goals: (i) using waste as an ingredient of the culture medium would solve the problem of their disposal and (ii) molecules of natural origin with high application potential in the food and cosmetic sectors would be produced. No substantial differences were observed in the qualitative composition of the C50 carotenoids obtained from growths performed with the different sugars; in all of them, BR was the major carotenoid produced.

The carotenoid extracts from *Hrd. utahensis* exhibited significant antioxidant power and anti-hyaluronidase activity, making them of great interest for applications in the skin care sector. In particular, the 2% fructose extract was a powerful HAase inhibitor and had strong ferric-reducing antioxidant power and superoxide scavenging capacity when compared to the positive controls BHT, ascorbic acid, and Trolox. In conclusion, the results obtained demonstrate that the extreme halophilic Archaeon *Hrd. utahensis* can be considered an effective microorganism to be used for the production of natural molecules with antioxidant activity.

In addition to the greater antioxidant power and HAase inhibitory activity, the 2% fructose growth provided the highest yields of carotenoids for both g of dry cells and liter of culture supernatant. However, the growth time was 3-fold longer than that required for glucose and xylose, making the production of BR and derivatives uncompetitive due to the greater expenditure of work hours and energy required to complete the process. To overcome this problem, solutions for improving the carotenoid yield in less time will be planned in the near future. The most suitable conditions to enhance the carotenoid production will be investigated by means of experimental methods such as response surface methodology. The influence of lower salt concentrations (osmotic stress) and hydrogen peroxide concentrations (oxidative stress) will be analyzed by this approach.

## Figures and Tables

**Table 1:** Growth parameters and carotenoid yields from *Hrd. utahensis* grown in M927 added with different sugars.

Carbon Source	Growth Time (Days)	OD (600 nm)	Dry Biomass (g/L)	Carotenoids (µg/g Dry Cell)	Carotenoids (µg/L)
0.2% Glucose	4	1.687	2.05	110.36 ± 5.05 ^a^	226.24 ± 14.64
1% Glucose	12.5	3.146	6.16	124.72 ± 7.32 ^a^	768.28 ± 63.77
2% Glucose	6	3.490	6.44	173.16 ± 11.48 ^b^	1115.15 ± 104.56
0.2% Fructose	23.5	0.506	1.71	453.43 ± 8.04 ^c^	775.36 ± 19.44
1% Fructose	24.5	1.940	3.10	403.05 ± 12.13 ^d^	1249.46 ± 53.18
2% Fructose	19.5	2.880	4.41	550.60 ± 7.91 ^e^	2428.15 ± 49.33
0.2% Xylose	5	1.116	1.80	266.36 ± 4.77 ^f^	479.45 ± 12.14
1% Xylose	6	3.360	5.48	275.42 ± 6.01 ^f^	1509.30 ± 46.58
2% Xylose	No growth	-	-	-	-

Values marked with different letters denote significant differences at *p* < 0.0001.

**Table 2:** List of carotenoids produced by *Hrd. utahensis* grown in M927 added with 2% glucose identified by HPLC/APCI-ITMS^n^.

	Peak ^a^	t_r_^b^ (min)	*Cis*λ_max_ ^c^ (nm)	λ_max_^c^ (nm)	% III/II	% A_B_/A_II_	[M + H]^+^ (*m*/*z*)	MS/MS Fragment Ion (*m*/*z*)	Molecular Formula	Carotenoid
2% Glucose	1	43.82	369.5, 387	467.5, 492.5, 525.5	46.5	14.4	741.5	723.4, 705.5, 687.5, 683.5, 665.5, 647.6, 635.5, 631.6, 629.6, 599.5, 591.5, 581.5, 563.6, 545.5, 537.6	C_50_H_76_O_4_	All-*trans*-bacterioruberin
	2	45.29	369, 386	459.5, 484, 516	42.0	20.9	741.5	723.4, 705.4, 687.4, 683.4, 665.3, 647.4, 635.3, 629.3, 599.6, 563.3, 549.3	C_50_H_76_O_4_	5-*cis,*9’-*cis*-Bacterioruberin
	3	45.78	368, 384	459, 483, 514.5	28.1	48	741.5	723.3, 705.4, 687.4, 665.3, 647.4, 631.3, 599.3	C_50_H_76_O_4_	Bacterioruberin isomer
	4	49.43	367.5, 386.5	463, 490, 522,5	54.8	12.7	741.5	723.3, 705.4, 687.4, 665.4, 647.4, 631.4, 599.4, 591.3, 563.5	C_50_H_76_O_4_	5-*cis*-Bacterioruberin
	5	52.54	370.5, 386.5	461, 486.5, 518.5	48.9	26.3	741.5	723.4, 705.4, 687.4, 683.3 665.4, 647.4, 563.4	C_50_H_76_O_4_	9-*cis*-Bacterioruberin
	6	54.39	369, 386	461, 486.5, 519	34.5	73.3	741.5	723.4, 705.4, 687.4, 683.3, 665.4, 647.4, 635.4, 631.3, 591.4, 563.4, 549.5	C_50_H_76_O_4_	13-*cis*-Bacterioruberin
	7	61.71	372, 387	468, 492.5, 525.5	47.6	14.1	723.5	705.4, 687.3, 647.4, 629.4, 617.3, 563.3, 549.4, 537.3, 523.5, 479.3	C_50_H_74_O_3_	All-*trans*-monoanhydrobacterioruberin
	8	66.50	387	461.5, 487.5, 518.5	44.9	10.9	723.5	705.4, 687.3, 647.3, 617.3, 537.5, 523.3, 563.3	C_50_H_74_O_3_	5-*cis*-Monoanhydrobacterioruberin
	9	68.71	373, 386	463, 484, 516	48.2	26.3	723.5	705.3, 687.5, 647.4, 629.4, 563.4, 549.5, 523.4, 507.3, 479.3	C_50_H_74_O_3_	9-*cis*-Monoanhydrobacterioruberin
	10	70.15	373.5, 387	459, 484, 517	44.6	63.9	723.5	705.3, 687.4, 647.3, 563.4, 549.3, 523.4, 507.3, 479.5	C_50_H_74_O_3_	13-*cis*-Monoanhydrobacterioruberin
	11	79.90	373, 389	465.5, 493, 526.5	49.9	16.2	705.5	687.4, 647.3, 599.5, 549.4, 537.4, 479.3	C_50_H_72_O_2_	All-*trans*-bisanhydrobacterioruberin

^a^ Peak number according to Figure 3A. ^b^ Reverse-phase HPLC retention time. ^c^ Maximum absorption obtained from UV/Vis spectra.

**Table 3:** List of carotenoids produced by *Hrd. utahensis* grown in M927 added with 2% fructose and 1% xylose.

	Peak ^a^	t_r_ ^b^ (min)	*Cis*λ_max_ ^c^ (nm)	λ_max_ ^c^ (nm)	% III/II	% A_B_/A_II_	Carotenoid
2% Fructose	1	44.98	369.5, 387	468, 492.5, 525	44.4	14.7	All-*trans*-bacterioruberin
	2	46.48	368, 385	460.5, 484.5, 516.5	41.2	21.3	5-*cis,*9’-*cis*-Bacterioruberin
	3	46.97	367, 384.5	458.5, 483.5, 514.5	30.5	48.8	Bacterioruberin isomer
	4	50.68	368.5, 386.5	463.5, 490, 522.5	54.3	13.1	5-*cis*-Bacterioruberin
	5	53.85	369, 386.5	461, 486.5, 518.5	47.3	25.7	9-*cis*-Bacterioruberin
	6	55.71	369, 386	461.5, 487, 519	33.1	67.5	13-*cis*-Bacterioruberin
	7	63.12	372, 389	465, 492, 524.5	41.2	14.7	All-*trans*-monoanhydrobacterioruberin
	8	67.95	387	467, 488, 515	22.1	11.2	5-*cis*-Monoanhydrobacterioruberin
1% Xylose	1	47.55	369, 387	469, 492.5, 525	41.9	15.7	All-*trans*-bacterioruberin
	2	49.04	368.5, 385	462, 485.5, 516.5	38.2	22.6	5-*cis*,9’-*cis*-Bacterioruberin
	3	49.48	367.5, 383.5	457, 483, 512	25.4	81.3	Bacterioruberin isomer
	4	53.19	368.5, 386	463.5, 490, 522.5	54.1	13.4	5-*cis-*Bacterioruberin
	5	56.28	369, 386.5	461.5, 486.5, 518.5	47	25.5	9-*cis*-Bacterioruberin
	6	58.09	369, 386	462, 487, 519.5	32.9	65.9	13-*cis*-Bacterioruberin
	7	65.34	371, 388.5	465, 493, 525.5	52.6	14.1	All-*trans*-monoanhydrobacterioruberin
	8	70.02	387	463, 486.5, 518.5	42.6	11.5	5-*cis*-Monoanhydrobacterioruberin
	9	72.16	373.5, 386	461.5, 487, 519	43.1	28.2	9-*cis*-Monoanhydrobacterioruberin
	10	73.62	373.5, 386	464, 484, 514.5	40.8	56.9	13-*cis*-Monoanhydrobacterioruberin
	11	83.05	389	467.5, 494.5, 526	58.6	16.2	All-*trans*-bisanhydrobacterioruberin

^a^ Peak number according to Figure 3B,C. ^b^ Reverse-phase HPLC retention time. ^c^ Maximum absorption obtained from UV/Vis spectra.

**Table 4:** FRAP assay.

*Hrd. utahensis* Extract	FRAP Response ^a^ (A_593nm_)	Ascorbic Acid Activity Equivalents ^b^ (µg)	Trolox Activity Equivalents ^b^ (µg)	BHT Activity Equivalents ^b^ (µg)
2% G	0.476 ± 0.018	3.028 ± 0.118 ^a^	4.789 ± 0.186 ^a^	183.077 ± 7.120 ^a^
2% F	0.854 ± 0.035	5.432 ± 0.221 ^b^	8.590 ± 0.350 ^b^	328.397 ± 13.391 ^b^
1% X	0.723 ± 0.018	4.601 ± 0.114 ^c^	7.277 ± 0.180 ^c^	278.205 ± 6.870 ^c^

^a^ FRAP values from 5 μg of *Hrd. utahensis* carotenoid extracts; ^b^ antioxidant activity equivalents; FRAP: Ferric Reducing Antioxidant Power; BHT: butylhydroxytoluene; 2% G = 2% glucose; 2% F = 2% fructose; 1% X = 1% xylose. Values in the same column marked with different letters denote significant differences at *p* < 0.001.

## Data Availability

Not applicable.

## Supplementary Materials

The following supporting information can be downloaded at: https://www.mdpi.com/article/10.3390/antiox12101840/s1, Table S1: Antioxidant activity of ascorbic acid, Trolox and BHT measured by FRAP assay, and related calibration curves.
