# Supplementary material for: Antioxidant Capacity of Carotenoid Extracts from the Haloarchaeon Halorhabdus utahensis
Source: Antioxidants (Basel). 2023 Oct 10;12(10):1840. doi: 10.3390/antiox12101840 (PMC10603985; doi:10.3390/antiox12101840)
Supplement: Supplementary file 1 [file antioxidants-12-01840-s001.zip › antioxidants-2634537-supplementary.pdf]

# Antioxidant Capacity of Carotenoid Extracts from the Haloarchaeon *Halorhabdus utahensis*

**Ismene Serino [1,2,†], Giuseppe Squillaci [2,†], Sara Errichiello [2], Virginia Carbone [3], Lidia Baraldi [4], Francesco La Cara [2] and Alessandra Morana [2,*]**

[1] Department of Experimental Medicine, University of Campania "Luigi Vanvitelli", Via Costantinopoli 16, 80138 Naples, Italy; ismene.serino@unicampania.it
[2] Research Institute on Terrestrial Ecosystems, National Research Council of Italy (CNR), Via Pietro Castellino 111, 80131 Naples, Italy; giuseppe.squillaci@iret.cnr.it (G.S.); errichiellosara.se@icloud.com (S.E.); francesco.lacara@cnr.it (F.L.C.)
[3] Institute of Food Sciences, National Research Council of Italy (CNR), Via Roma 64, 83100 Avellino, Italy; virginia.carbone@cnr.it (V.C.)
[4] Institute of Experimental Endocrinology and Oncology "Gaetano Salvatore", National Research Council of Italy (CNR), Via S. Pansini 5, 80131 Naples, Italy; lidia.baraldi@cnr.it
* Correspondence: alessandra.morana@cnr.it
† These authors contributed equally to this work.

**Table S1.** Antioxidant activity of ascorbic acid, Trolox and BHT measured by FRAP assay, and related calibration curves.

| **Ascorbic acid** (μg) | **$A_{593nm}$** |
|---|---|
| 0.25 | 0.042 ± 0.004 |
| 0.5 | 0.074 ± 0.003 |
| 1.0 | 0.150 ± 0.006 |
| 2.0 | 0.316 ± 0.005 |
| 5.0 | 0.787 ± 0.008 |

Calibration curve: $A_{593nm}$ = 0.1572 μg  $r^2$ = 0. 9998

| **BHT** (μg) | **$A_{593nm}$** |
|---|---|
| 5.00 | 0.017 ± 0.002 |
| 50.0 | 0.116 ± 0.021 |
| 100.0 | 0.240 ± 0.020 |
| 200.0 | 0.535 ± 0.052 |

Calibration curve: $A_{593nm}$ = 0.0026 μg  $r^2$ = 0. 9945

| **Trolox** (μg) | **$A_{593nm}$** |
| --- | --- |
| 1.0 | 0.066 ± 0.006 |
| 2.5 | 0.224 ± 0.010 |
| 5.0 | 0.49 ± 0.028 |
| 7.0 | 0.715 ± 0.054 |

Calibration curve: $A_{593nm}$ = 0.0994 μg  $r^2$ = 0. 9914
